# Biochemical phenotypes of acute kidney injury and their association with major adverse kidney events

**DOI:** 10.1080/0886022X.2026.2620162

**Published:** 2026-01-27

**Authors:** Pablo Maggiani-Aguilera, Guillermo Navarro-Blackaller, Pedro A. Rodriguez-Peña, Yohevana García-Barrón, Edgar Saldaña-Rocha, Jose A. Gómez, Jonathan S. Chávez-Iñiguez

**Affiliations:** a Internal Medicine Department, Hospital General de Mazatlan, Sinaloa, Mexico; b Nephrology Service, Hospital Civil de Guadalajara “Fray Antonio Alcalde”, Guadalajara, Jalisco, Mexico; c University of Guadalajara Health Sciences Center, Guadalajara, Jalisco, Mexico; d Nephrology Service, Hospital General de Mazatlan, Sinaloa, Mexico

**Keywords:** Acute kidney injury, phenotypes, MAKE, major adverse kidney events, mortality, kidney replacement therapy

## Abstract

Acute kidney injury (AKI) is a heterogeneous syndrome in which conventional stratification based on serum creatinine and urine output may fail to capture the full spectrum of underlying metabolic disturbances. In a retrospective cohort study of hospitalized adults with AKI admitted to Hospital General de Mazatlán between January 2022 and July 2025, we sought to identify biochemical phenotypes associated with major adverse kidney events (MAKE). Demographic, clinical, and biochemical variables, including urea, creatinine, calcium, phosphorus, potassium, hemoglobin, albumin, and pH, were standardized using z-scores and clustered using k-means analysis. The primary outcome was MAKE, defined as in-hospital mortality or new requirement for kidney replacement therapy (KRT). Among 797 patients, three distinct biochemical phenotypes were identified: Phenotype 1, characterized by relatively preserved biochemical parameters; Phenotype 2, defined by anemia and hypocalcemia; and Phenotype 3, marked by severe metabolic derangements. Phenotype 3 exhibited the most severe kidney dysfunction and metabolic instability, including anemia, hyperkalemia, hyperphosphatemia, hypocalcemia, and acidosis, and was strongly associated with MAKE (odds ratio [OR] 3.39, 95% confidence interval [CI] 2.12–5.40; *p* < 0.001), in-hospital mortality (OR 2.47, 95% CI 1.48–4.12; *p* < 0.001), and initiation of KRT (OR 5.80, 95% CI 1.60–21.12; *p* = 0.007). Phenotype 2 was more frequently observed in women and was associated with AKI stage 3, gastrointestinal bleeding, and malignancy, but was not independently associated with MAKE. Biochemical phenotyping identified clinically meaningful AKI subgroups and highlighted a high-risk phenotype characterized by profound metabolic instability and adverse kidney outcomes.

## Introduction

Acute kidney injury (AKI) is a syndrome that occurs in up to 35% of hospitalized patients [1] and is associated with high morbidity and mortality, prolonged hospital stays, and increased healthcare costs [2]. Its clinical and pathophysiological heterogeneity makes it difficult to predict disease trajectory, outcomes, and to individualize therapeutic strategies [3]. The traditional stratification proposed by KDIGO provides useful prognostic information based on changes in serum creatinine and urine output; however, it is incomplete, as it does not capture other metabolic alterations frequently observed in clinical settings of patients with this syndrome [4], such as electrolyte abnormalities [5–7] acid–base imbalances, and other hematological biomarkers [8]. During AKI, routinely available clinical variables such as hemoglobin, calcium, phosphorus, potassium, and pH may indirectly reflect the systemic impact of the syndrome [9]. Incorporating these parameters into the initial evaluation could provide a broader view of the systemic spectrum of AKI, allowing patients to be phenotyped into more stratified profiles in which therapeutic interventions could be better targeted and potentially more effective [10,11]. The use of unsupervised approaches such as cluster analysis has enabled the identification of AKI subphenotypes, reducing the need for kidney replacement therapy (KRT) and mortality [12]. Given the growing interest in the early identification and classification of patients with AKI and the existing knowledge gap in this field, our objective was to identify biochemical phenotypes in hospitalized patients with AKI by integrating multiple routinely used biochemical variables. This approach aimed to achieve a real-time risk stratification and to evaluate their association with major adverse kidney events (MAKE), thereby guiding more focused clinical and therapeutic efforts.

## Methods

### Study design and patient population

In this retrospective cohort study conducted between January 2022 and July 2025, we enrolled patients admitted to the Mazatlán General Hospital, patients were in the intensive care units and in the general hospital wards. We focused on patients diagnosed with AKI according to KDIGO [9]. Patients aged ≥18 years with complete biochemical and clinical data at hospital admission were included. Exclusion criteria were uncertain diagnosis of AKI, chronic kidney disease (CKD) without AKI, prior chronic kidney replacement therapy (KRT), or incomplete records for the analyzed variables. Eligible patients were assessed at the time of their initial AKI diagnosis and subsequently followed during clinical visits. Demographic variables (age, sex), comorbidities, causes of admission, biochemical parameters (urea, serum creatinine, calcium, phosphorus, potassium, hemoglobin, albumin), and major adverse kidney events (MAKE) were collected. MAKE was defined according to the National Institute of Diabetes and Digestive and Kidney Disease workgroup for the study of AKI [13], and includes two clinically meaningful short-term, patient-centered outcomes: death and new requirement for KRT [14]. AKI was classified into stages 1, 2, and 3 according to KDIGO criteria. AKI on CKD was defined as the presence of a prior history of CKD with evidence of AKI [9]. The indications for KRT included diuretic-resistant fluid overload, severe hyperkalemia, severe metabolic acidosis, and uremic complications such as encephalopathy, pericarditis, or seizures [15,16]. KRT was provided using intermittent hemodialysis, with prescription tailored to the patient’s clinical needs. In accordance with the 31st Acute Disease Quality Initiative group recommendations on the design of cohorts and clinical trials in AKI, we aimed to explore the clinical phenotyping of these patients [17].

### Study objectives

The primary objective was to evaluate the association between AKI phenotypes and MAKE. Secondary objectives included assessing the association of AKI phenotypes with the individual components of MAKE (KRT and mortality), as well as with AKI progression.

The primary endpoint, MAKE was assessed during hospitalization. Mortality was adjudicated as death occurring prior to discharge. Initiation of KRT was defined by the documented commencement of hemodialysis for standard clinical indications. Follow-up was limited to the duration of hospitalization, with patients censored at the time of discharge.

### Ethical considerations

The protocol was reviewed and approved by the Local Research and Health Ethics Committee of the Hospital General de Mazatlán (CEI-2025-38). In adherence to the ethical standards of the Declaration of Helsinki, informed consent was not required for this study. The study protocol was designed in accordance with the Strengthening the Reporting of Observational Studies in Epidemiology (STROBE) guidelines [18] and the REporting of studies Conducted using Observational Routinely Collected Health Data (RECORD) statement [19].

### Statistical analysis

Cluster analysis using the k-means algorithm was performed on standardized biochemical variables to derive distinct phenotypes. To explore their clinical relevance, multivariable logistic regression models were subsequently fitted to estimate odds ratios (OR) with 95% confidence intervals (95% CI), adjusting for covariates that demonstrated significance in the bivariate analysis. The study population comprised all eligible patients (*n* = 797). Given the observed distribution of phenotypes and predictors, the study had greater than 80% power to detect an OR ≥ 2.0 for the comparison of Phenotype 2 versus Phenotype 1, and an OR ≥ 1.8 for the comparison of Phenotype 3 versus Phenotype 1, at a two-sided alpha level of 0.05. Statistical significance was defined as *p* < 0.05. To further assess the robustness and consistency of the association between biochemical AKI phenotypes and MAKE, we conducted a series of prespecified sensitivity and stratified analyses. These included separate multivariable logistic regression models for each component of MAKE (mortality and initiation of KRT), stratification according to baseline CKD status and AKI etiology (septic vs non-septic AKI), as well as formal interaction testing between phenotype and CKD status and between phenotype and AKI etiology. All analyses were conducted using R software (R Foundation for Statistical Computing, Vienna, Austria).

## Results

**Figure 1.**
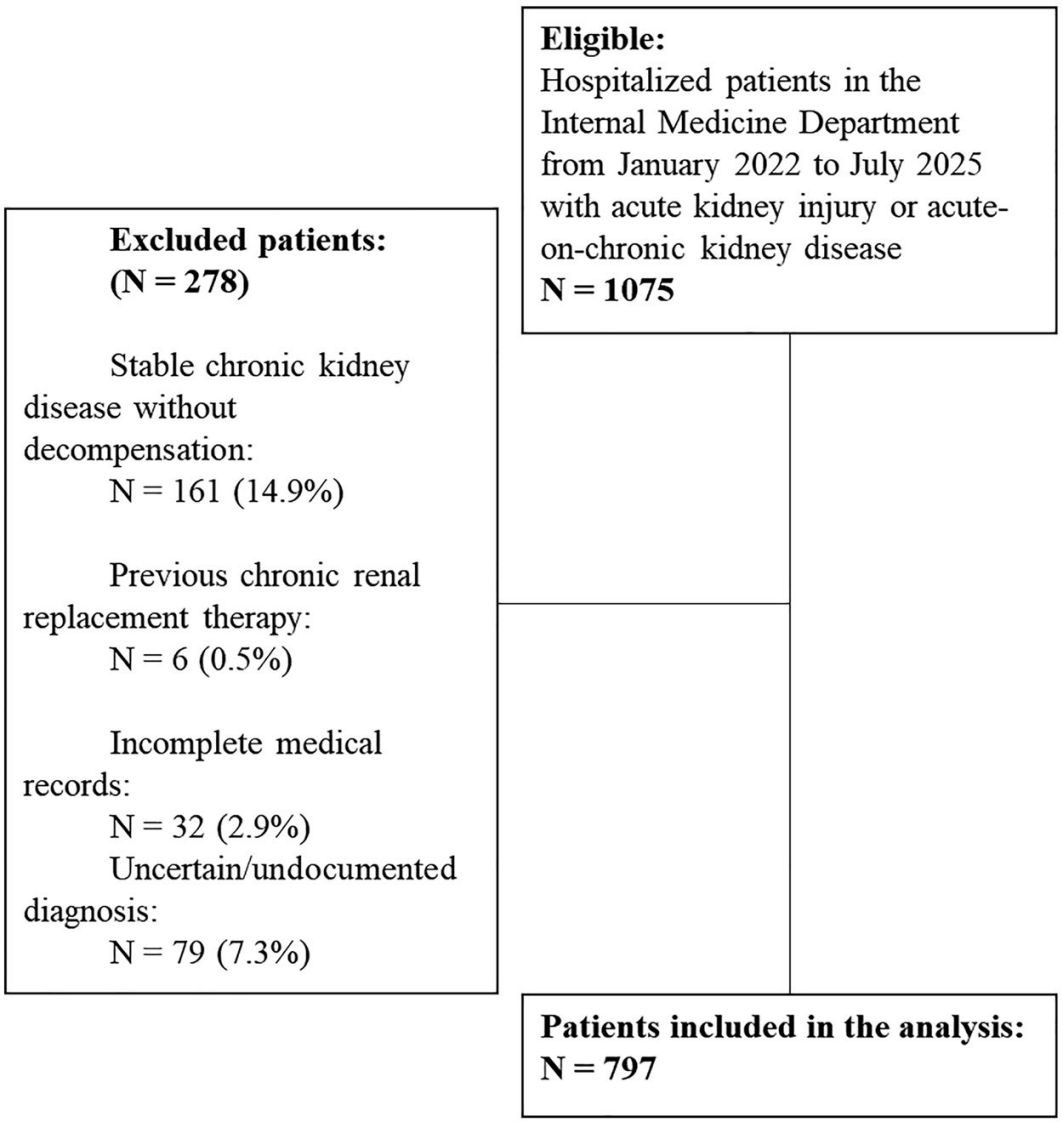
Flow diagram of the study population.

**Figure 2.**
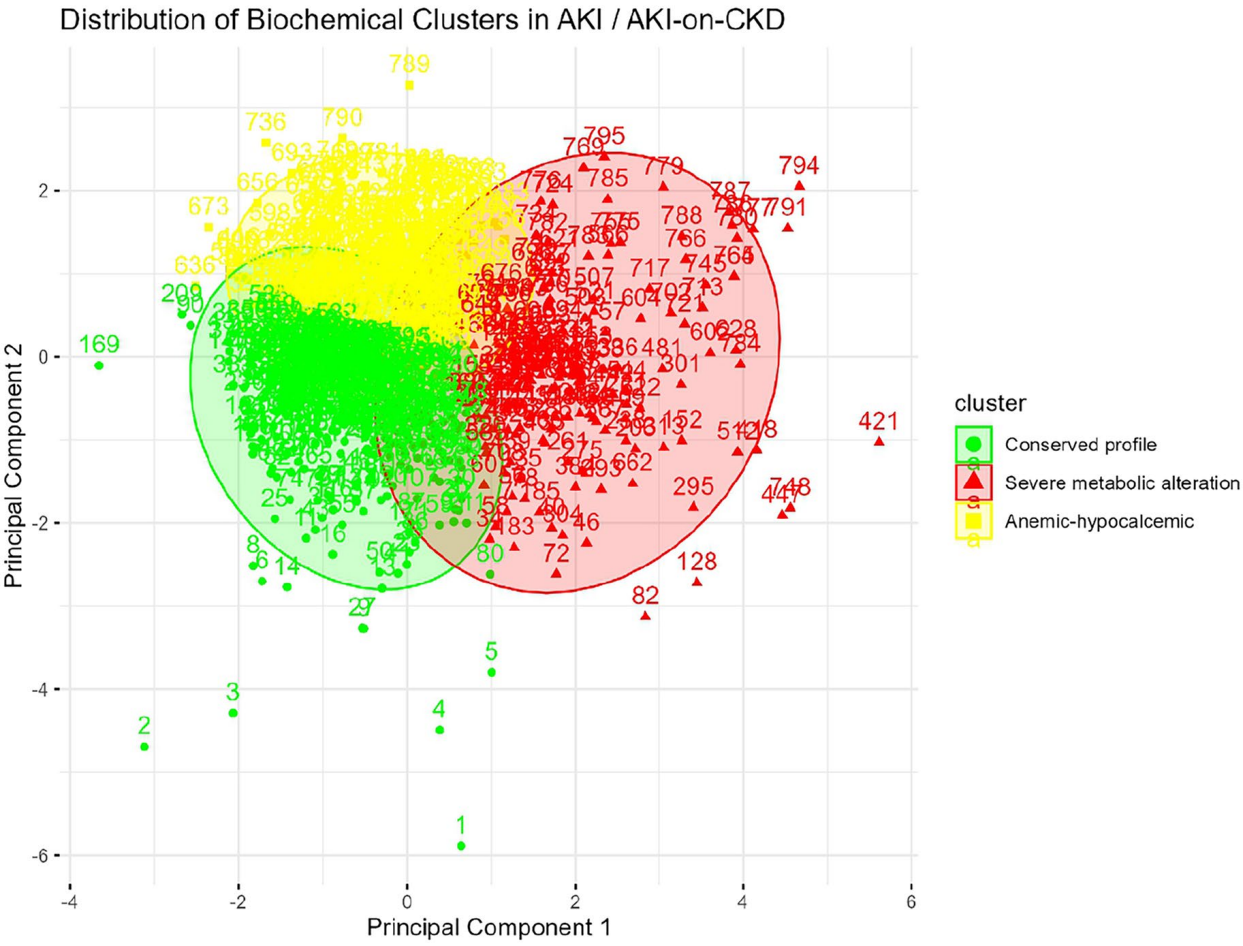
Scatterplot of clusters in standardized space with overlaid normal ellipses. Principal component analysis showing the three clusters derived from k-means. Phenotype 1 (green, *n* = 341) exhibited a preserved biochemical profile; Phenotype 2 (yellow, *n* = 290) showed an anemic–hypocalcemic pattern with mild mineral abnormalities; and Phenotype 3 (red, *n* = 166) was defined by severe metabolic disturbances (anemia, hypocalcemia, hyperphosphatemia, hyperkalemia, and acidosis). Clear separation, particularly for Phenotype 2, supports the clinical validity of the classification.

**Figure 3.**
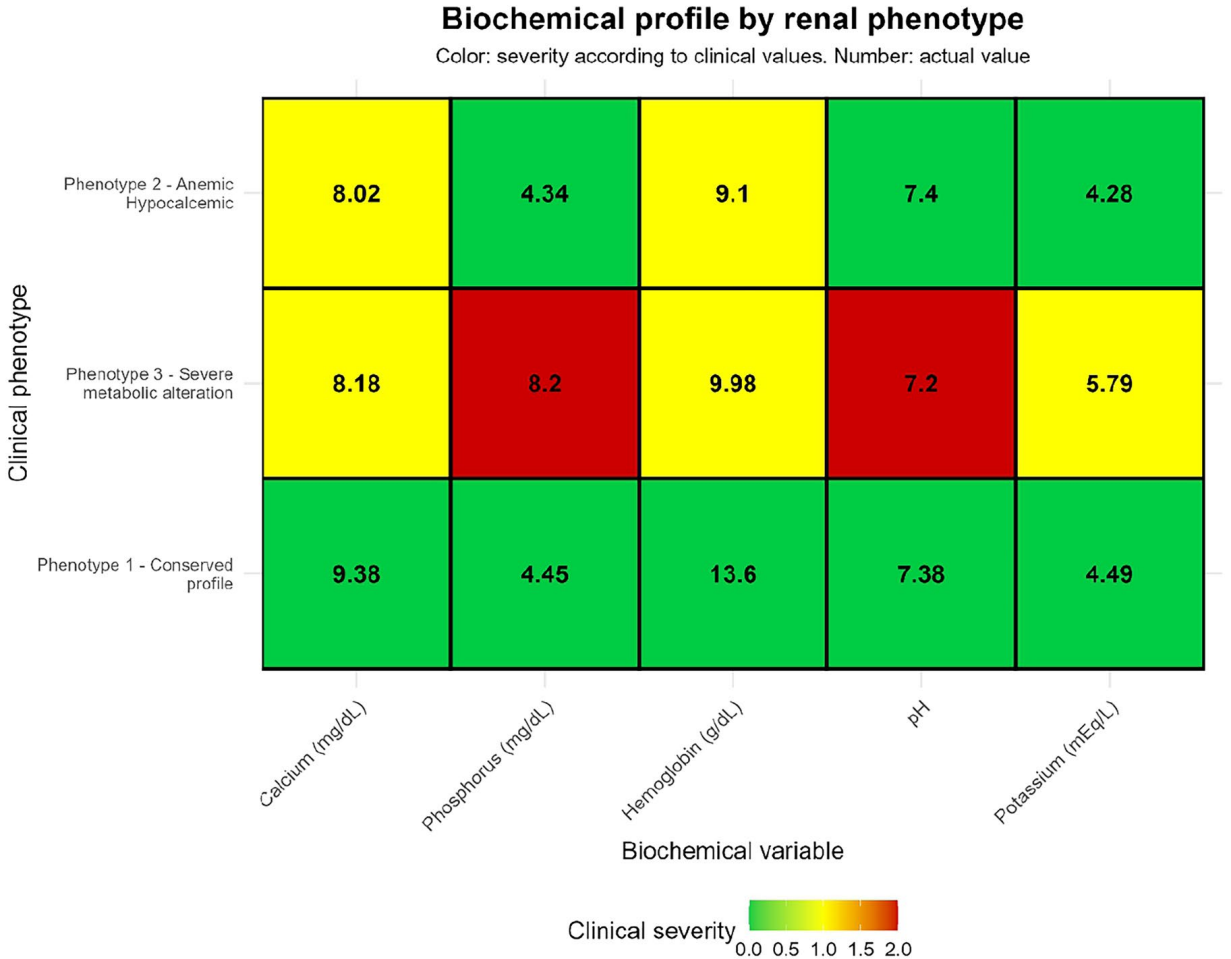
Heatmap of the biochemical profile by phenotype. Mean values and gradient of clinical alteration.

During the cohort period, a total of 1,075 patients were hospitalized in the Department of Internal Medicine. Of these, 278 were excluded, leaving 797 patients who fulfilled the inclusion criteria and were included in the analysis, as depicted in the flow chart (Figure 1). Using admission variables as hemoglobin, calcium, pH, phosphorus, and potassium a k-means clustering analysis with z-score standardization was performed. The optimal number of clusters was identified through the elbow method, and the final model (set.seed = 123, centers = 3, nstart = 25) classified the study population into three distinct phenotypes. Phenotype 1, representing a preserved biochemical profile, comprised 341 patients; Phenotype 2, defined by anemia and hypocalcemia, included 290 patients; and Phenotype 3, characterized by severe metabolic disturbances, encompassed 166 patients. The separation of these clusters is illustrated in multivariate space (Figure 2), highlighting the distinct biochemical patterns of each group. A clinical heatmap (Figure 3) visually summarized these biochemical profiles, clearly depicting the gradient of alterations in relation to established clinical thresholds. The cohort was stratified into three distinct phenotypes with marked clinical and biochemical differences, Table 1. Patients in Phenotype 1 (conserved profile, *N* = 341) were older on average (65.4 ± 16.8 years) and had a higher proportion of men (58.4%). In contrast, Phenotype 3 (severe metabolic disturbance, *N* = 166) included younger individuals (58.9 ± 15.8 years), predominantly male (62.7%), and was characterized by profound metabolic derangements, including markedly elevated creatinine (7.48 ± 7.90 mg/dL), hyperkalemia (5.79 ± 1.13 mmol/L), hyperphosphatemia (8.20 ± 2.51 mg/dL), metabolic acidosis (pH 7.20 ± 0.15), and severe anemia (hemoglobin 9.98 ± 2.95 g/dL). Phenotype 2 (anemic–hypocalcemic, *N* = 290) was distinguished by a predominance of women (52.4%) and by pronounced anemia (hemoglobin 9.10 ± 2.15 g/dL) and hypocalcemia (8.02 ± 0.88 mg/dL), with moderately impaired kidney function (creatinine 3.28 ± 3.92 mg/dL).

**Table 1.** Distribution of the hospitalized population based on phenotypes.

Variable	Phenotype 1 – preserved profile (*N* = 341)	Phenotype 2 – anemic–hypocalcemic (*N* = 290)	Phenotype 3 – severe metabolic alteration (*N* = 166)	*p* value
Age	65.40 ± 16.75	61.90 ± 17.20	58.95 ± 15.80	0.0001*
Female	142 (41.6%)	152 (52.4%)	62 (37.3%)	0.0019*
Male	199 (58.4%)	138 (47.5%)	104 (62.6%)	0.0019*
Comorbidities				
Type 2 diabetes mellitus (T2DM)	139 (40.8%)	128 (44.1%)	72 (43.4%)	0.6734
Hypertension (HTN)	184 (54.1%)	147 (50.7%)	87 (52.4%)	0.6913
Heart failure (HF)	63 (18.5%)	38 (13.1%)	25 (15.2%)	0.1711
Hypothyroidism	10 (2.9%)	13 (4.5%)	5 (3%)	0.5309
Chronic kidney disease (CKD)	81 (23.7%)	67 (23.1%)	46 (27.7%)	0.6891*
Previous myocardial infarction (MI)	9 (2.6%)	12 (4.2%)	4 (2.4%)	0.4617
Previous stroke	24 (7%)	12 (4.1%)	4 (2.4%)	0.0561
Substance use disorders	92 (27.1%)	66 (23%)	38 (23%)	0.4122
Admission diagnoses				
Pancreatitis	3 (0.9%)	1 (0.3%)	1 (0.6%)	0.8476
Urinary tract infection (UTI)	69 (20.2%)	76 (26.2%)	27 (16.3%)	0.0333*
Neuroinfection	3 (0.9%)	0 (0%)	0 (0%)	0.2375
Acute coronary syndrome (ACS)	26 (7.6%)	10 (3.4%)	4 (2.4%)	0.0127*
Acute heart failure	55 (16.1%)	27 (9.3%)	20 (12%)	0.0362*
Gastrointestinal bleeding	17 (5%)	40 (13.8%)	13 (7.8%)	0.0005*
COPD exacerbation	32 (9.4%)	14 (4.8%)	7 (4.2%)	0.0268*
Community-acquired pneumonia (CAP)	68 (19.9%)	28 (9.7%)	19 (11.4%)	0.0006*
Hypertensive emergency	15 (4.4%)	20 (6.9%)	12 (7.2%)	0.2964
Unspecified neoplasm	6 (1.8%)	16 (5.5%)	5 (3%)	0.0325*
Decompensated cirrhosis	21 (6.2%)	15 (5.2%)	6 (3.6%)	0.4829
Stroke	26 (7.6%)	15 (5.2%)	8 (4.8%)	0.3206
Seizures	16 (4.7%)	4 (1.4%)	4 (2.4%)	0.0456*
Septic shock	43 (12.6%)	51 (17.6%)	26 (15.7%)	0.2128
Other shock	10 (2.9%)	17 (5.9%)	12 (7.2%)	0.0690
Tuberculosis	8 (2.3%)	15 (5.2%)	4 (2.4%)	0.1087
Withdrawal syndrome	2 (0.6%)	0 (0%)	0 (0%)	0.6882
Drug intoxication/overdose	6 (1.8%)	0 (0%)	4 (2.4%)	0.0198*
Diabetic ketoacidosis (DKA)	15 (4.4%)	7 (2.4%)	13 (7.8%)	0.0250*
Purpura	1 (0.3%)	2 (0.7%)	0 (0%)	0.6047
Systemic lupus erythematosus (SLE)	0 (0%)	2 (0.7%)	1 (0.6%)	0.3202
Leukemia	0 (0%)	1 (0.3%)	0 (0%)	0.5721
Laboratory variables				
Hemoglobin (g/dL)	13.60 ± 2.03	9.10 ± 2.15	9.98 ± 2.95	0.0000*
Urea (mg/dL)	97.05 ± 64.66	123.64 ± 92.11	198.98 ± 120.09	0.0000*
Creatinine (mg/dL)	2.50 ± 2.13	3.28 ± 3.92	7.48 ± 7.90	0.0000*
Glucose (mg/dL)	205.02 ± 171.90	174.86 ± 132.06	188.81 ± 207.68	0.0023*
Potassium (mEq/L)	4.49 ± 0.88	4.28 ± 0.89	5.79 ± 1.13	0.0000*
Calcium (mg/dL)	9.38 ± 1.27	8.02 ± 0.88	8.18 ± 1.32	0.0000*
Phosphorus (mg/dL)	4.45 ± 1.53	4.34 ± 1.49	8.20 ± 2.51	0.0000*
pH	7.38 ± 0.11	7.40 ± 0.09	7.20 ± 0.15	0.0000*
AKI stages				
AKI KDIGO stage 1	195 (57.2%)	125 (43.1%)	25 (15.1%)	0.0000*
AKI KDIGO stage 2	78 (22.9%)	59 (20.3%)	32 (19.3%)	0.0000*
AKI KDIGO stage 3	68 (19.9%)	106 (36.6%)	109 (65.7%)	0.0000*
Length of stay (days)	6.14 ± 5.66	7.08 ± 6.80	6.65 ± 6.36	0.2113
Hemodialysis	3 (0.9%)	4 (1.4%)	21 (12.7%)	0.0000*
Death	75 (22%)	82 (28.3%)	55 (33.1%)	0.0207*
MAKE	77 (22.6%)	85 (29.3%)	91 (45.2%)	0.0000*

MAKE (major adverse kidney events) is a composite of new requirement for hemodialysis or death.

*p < 0.05.

Comorbidity distribution was relatively balanced across phenotypes, although diabetes mellitus and hypertension were highly prevalent overall (approximately 40–50%), while cardiovascular disease and prior cerebrovascular events occurred less frequently. Admission diagnoses varied significantly: acute coronary syndrome and pneumonia were more common in Phenotype 1, while acute heart failure and gastrointestinal bleeding were more frequent in Phenotype 3, and urinary tract infections were most prevalent in Phenotype 2. Importantly, the severity of AKI followed a distinct pattern: KDIGO stage 3 was predominant in Phenotype 3 (65.7%), while stage 1 was more frequent in Phenotype 1 (57.2%) and stage distribution was more heterogeneous in Phenotype 2.

Clinical outcomes reflected these differences. KRT was primarily required in Phenotype 3 (12.7%), compared with less than 2% in the other groups. Mortality was also highest in Phenotype 3 (33.1%), followed by Phenotype 2 (28.3%) and Phenotype 1 (22%). These findings highlight that Phenotype 3 represents the subgroup with the most severe metabolic disturbances, advanced kidney dysfunction, and worst prognosis, while Phenotype 1 retained a relatively conserved profile with lower dialysis requirements and mortality, and Phenotype 2 showed an intermediate risk pattern driven by anemia and hypocalcemia.

### Multinomial model: factors associated with each phenotype

**Figure 4.**
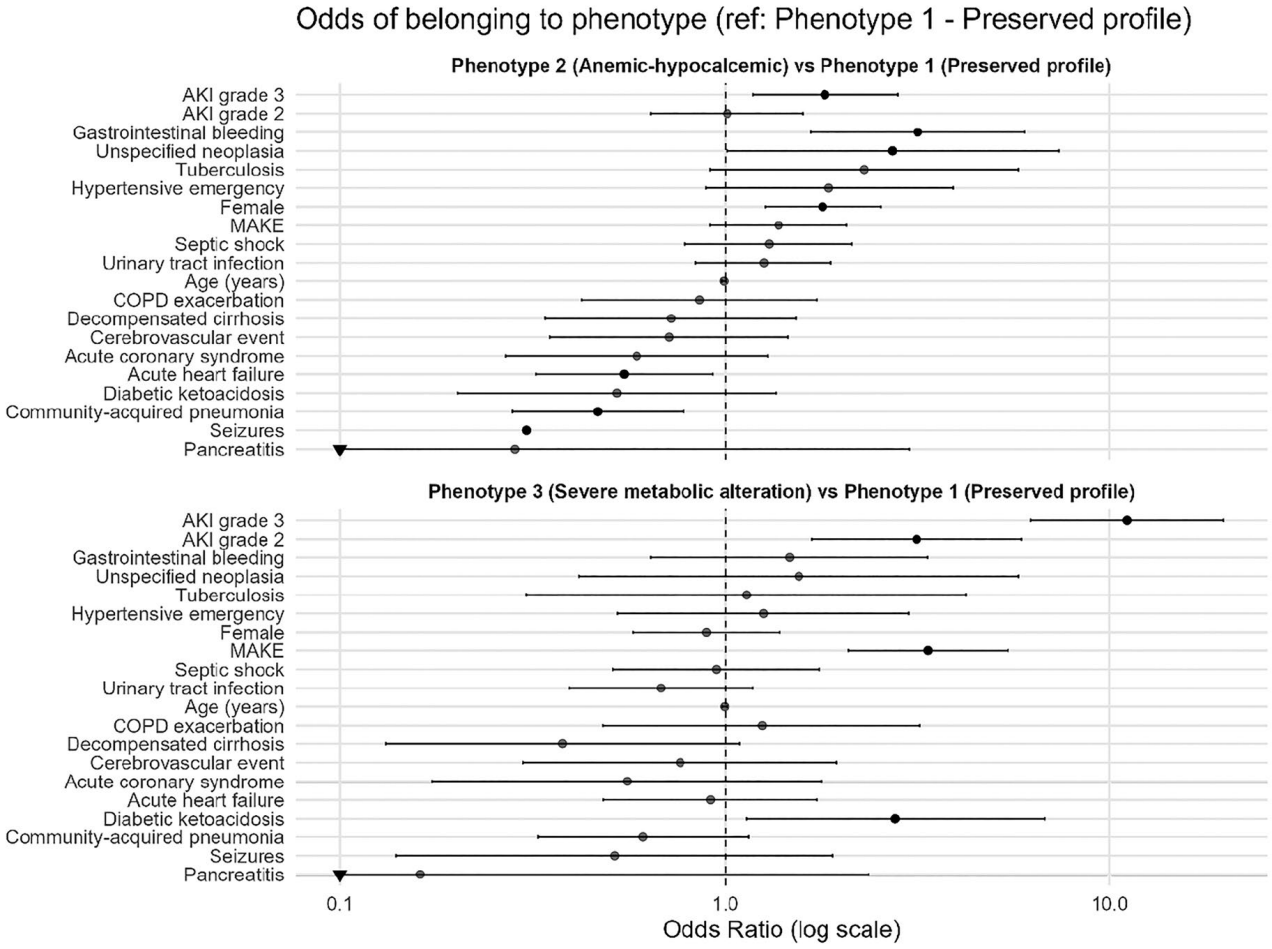
Forest plot illustrating associations from the multinomial regression model.

In the multinomial regression model, with Phenotype 1 (preserved profile) as the reference category, Phenotype 3 (severe metabolic disturbance) emerged as the group most strongly associated with adverse outcomes. This phenotype was independently linked to the primary outcome MAKE (OR 3.39; 95% CI 2.12–5.40; *p* < 0.001), and carried a higher probability of requiring KRT (OR 5.80; 95% CI 1.60–21.12; *p* = 0.007) and in-hospital mortality (OR 2.47; 95% CI 1.48–4.12; *p* < 0.001). Additionally, Phenotype 3 was associated with more advanced stages of AKI, including stage 2 (OR 3.27; 95% CI 1.75–6.11; *p* < 0.001) and stage 3 (OR 10.67; 95% CI 5.96–19.10; *p* < 0.001), as well as with diabetic ketoacidosis (OR 2.51; 95% CI 1.03–6.10; *p* = 0.043). No significant associations were observed with demographic characteristics, septic shock, community-acquired pneumonia, or other comorbidities (Tables 2 and 3 and Figure 4).

**Table 2.** Multivariable analysis: association of clinical variables with membership in biochemical phenotypes (reference: Phenotype 1 – Preserved profile).

Comparison	Variable	OR	95% CI	*p*
Phenotype 3 – Severe metabolic alteration vs Phenotype 1	Age (years)	1.00	0.98–1.02	0.716
	Female	0.93	0.60–1.45	0.759
	In-hospital mortality	2.47	1.48–4.12	<0.001*
	Hemodialysis	5.80	1.60–21.12	0.007*
	AKI stage 2	3.27	1.75–6.11	<0.001*
	AKI stage 3	10.67	5.96–19.10	<0.001*
	Septic shock	0.98	0.53–1.81	0.954
	Pancreatitis	0.13	0.01–1.36	0.170
	Urinary tract infection	0.64	0.37–1.11	0.121
	Acute coronary syndrome	0.55	0.17–1.77	0.319
	Acute heart failure	0.87	0.45–1.69	0.678
	Gastrointestinal bleeding	1.54	0.66–3.59	0.320
	COPD exacerbation	1.24	0.48–3.20	0.659
	Community-acquired pneumonia (CAP)	0.62	0.33–1.16	0.134
	Hypertensive emergency	1.16	0.48–2.77	0.745
	Unspecified neoplasm	2.52	0.66–9.63	0.174
	Decompensated cirrhosis	0.44	0.15–1.27	0.123
	Stroke	0.95	0.39–2.29	0.920
	Seizures	0.86	0.25–2.95	0.812
	Tuberculosis	1.25	0.34–4.53	0.739
	Diabetic ketoacidosis	2.51	1.03–6.10	0.043*
Phenotype 2 – Anemic–hypocalcemic vs Phenotype 1	Age (years)	0.99	0.98–1.00	0.128
	Female	1.94	1.36–2.76	<0.001*
	In-hospital mortality	1.41	0.92–2.15	0.113
	Hemodialysis	0.80	0.17–3.67	0.784
	AKI stage 2	1.04	0.66–1.65	0.854
	AKI stage 3	1.94	1.25–2.99	0.003*
	Septic shock	1.18	0.71–1.97	0.518
	Pancreatitis	0.29	0.03–2.58	0.306
	Urinary tract infection	1.26	0.84–1.89	0.266
	Acute coronary syndrome	0.59	0.27–1.30	0.191
	Acute heart failure	0.53	0.31–0.91	0.021*
	Gastrointestinal bleeding	3.50	1.82–6.70	<0.001*
	COPD exacerbation	0.86	0.43–1.72	0.685
	Community-acquired pneumonia (CAP)	0.46	0.28–0.78	0.003*
	Hypertensive emergency	1.86	0.89–3.87	0.101
	Unspecified neoplasm	3.16	1.09–9.17	0.034*
	Decompensated cirrhosis	0.69	0.33–1.45	0.340
	Stroke	0.71	0.35–1.43	0.355
	Seizures	0.45	0.15–1.34	0.150
	Tuberculosis	2.30	0.91–5.84	0.078
	Diabetic ketoacidosis	0.50	0.19–1.34	0.156

*p < 0.05.

**Table 3.** Adjusted odds ratios for mortality, initiation of hemodialysis, and MAKE according to clinical phenotype (reference: Phenotype 1 – Preserved profile).

Comparison (vs phenotype 1 – reference)	In-hospital mortality OR (95% CI)	*p*	Hemodialysis OR (95% CI)	*p*	MAKE OR (95% CI)	*p*
Phenotype 3 – Severe metabolic alteration	2.47 (1.48–4.12)	<0.001	5.80 (1.60–21.12)	0.007	3.39 (2.12–5.40)	<0.001
Phenotype 2 – Anemic–hypocalcemic	1.41 (0.92–2.15)	0.113	0.80 (0.17–3.67)	0.784	1.38 (0.91–2.08)	0.13

Adjusted odds ratios (95% CI) derived from three independent multivariable models: mortality, hemodialysis, and MAKE. Each model included the same clinical and laboratory covariates; outcomes are displayed in parallel solely to facilitate comparison.

In contrast, Phenotype 2 (anemic–hypocalcemic) did not show associations with MAKE (OR 1.38; 95% CI 0.91–2.08; *p* = 0.13), KRT (OR 0.80; 95% CI 0.17–3.67; *p* = 0.784), or in-hospital mortality (OR 1.41; 95% CI 0.92–2.15; *p* = 0.113). Nevertheless, it was more frequently observed in women (OR 1.94; 95% CI 1.36–2.76; *p* < 0.001) and was associated with AKI stage 3 (OR 1.94; 95% CI 1.25–2.99; *p* = 0.003), gastrointestinal bleeding (OR 3.50; 95% CI 1.82–6.70; *p* < 0.001), and neoplasia (OR 3.16; 95% CI 1.09–9.17; *p* = 0.034). Interestingly, this phenotype also demonstrated a lower probability of acute heart failure (OR 0.53; 95% CI 0.31–0.91; *p* = 0.021) and community-acquired pneumonia (OR 0.46; 95% CI 0.28–0.78; *p* = 0.003) (Tables 2 and 3 and Figure 4). Sensitivity and stratified analyses yielded consistent results across subgroups. Phenotype 3 remained independently associated with MAKE in patients with AKI and in those with AKI on preexisting CKD, as well as in septic and non-septic AKI. Formal interaction testing showed no statistically significant interaction between phenotype and CKD status or AKI etiology, indicating that the association between phenotype 3 and adverse outcomes was broadly consistent across clinical contexts (Supplementary Appendix).

## Discussion

**Figure 5.**
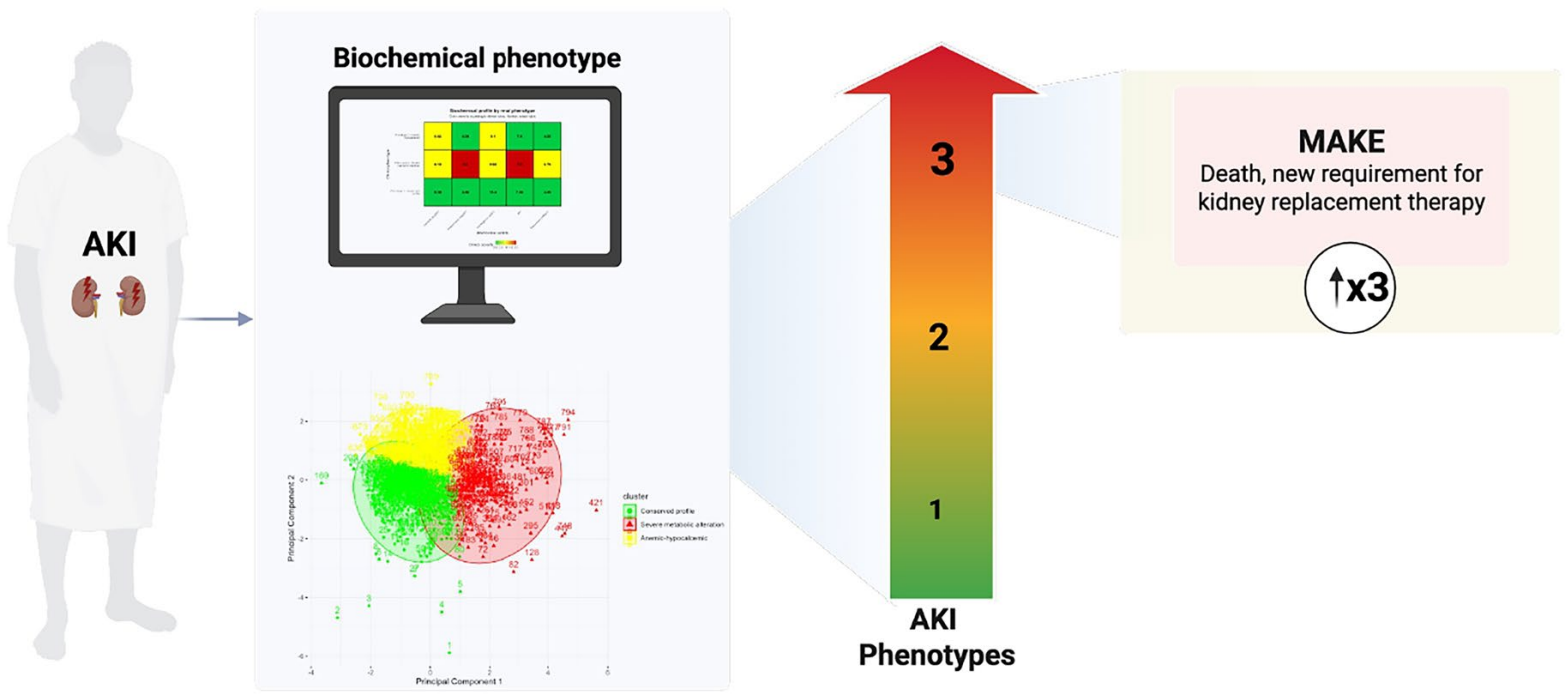
Central figure.

In this cohort of hospitalized patients with AKI, we identified three distinct phenotypes using routinely available clinical and biochemical variables, enabling the stratification of patients according to their risk of developing MAKE (Figure 5)

These phenotypes demonstrated important differences that help explain their divergent clinical trajectories. Phenotype 1 corresponded to a preserved profile, characterized by metabolic parameters close to the normal range and more favorable clinical outcomes. Phenotype 3 reflected greater AKI severity and a pattern of profound metabolic disturbance, combining metabolic acidosis, hyperkalemia, hyperphosphatemia, hypocalcemia, and anemia, and was strongly associated with high mortality and a markedly increased need for KRT. Finally, Phenotype 2 exhibited a predominantly anemic–hypocalcemic pattern, without the severity observed in Phenotype 3, but with notable associations with specific comorbidities and a potential risk for disease progression.

From the perspective of clinical outcomes, Phenotype 3, when compared with Phenotype 1, represented the group with the poorest prognosis. It was associated with more than a threefold increased risk of MAKE, a fivefold higher likelihood of requiring KRT, and a twofold increase in in-hospital mortality, in addition to a greater probability of progression to AKI stage 3.

The higher prevalence of chronic comorbidities and admission diagnoses observed in Phenotypes 1 and 2, compared with 3, may appear counterintuitive at first glance. This finding may reflects differences in the clinical context and timing of AKI rather than a lower disease burden. Phenotype 3 presented with advanced AKI and severe metabolic derangements driven by acute systemic insults, which may dominate the clinical picture and limit the relative contribution of chronic comorbidities. In contrast, Phenotypes 1–2 represent patients with greater chronic cardiovascular and pulmonary comorbidity, in whom AKI may develop during prolonged hospitalization or as a complication of medical decompensation. These observations support the concept that biochemical phenotypes capture distinct pathophysiological pathways of AKI, rather than simply reflecting comorbidity load, and help explain the differing outcome profiles observed across phenotypes.

Efforts to phenotype patients with AKI have been attempted through different approaches. Plasma and urinary biomarker analyses combined with clinical variables have been used to identify molecular subphenotypes of AKI, describing two major subgroups that exhibit distinct risks of long-term MAKE, independent of AKI severity [12]. Moreover, these subphenotypes have demonstrated differential responses to vasopressin in the context of septic shock. In the field of cardiac surgery–associated AKI, trajectory modeling of serum creatinine has enabled the identification of up to 12 phenotypes, four of which were associated with increased mortality risk [20]. Similarly, the analysis of AKI recovery patterns in critically ill patients has revealed five recovery phenotypes, with marked differences in one-year survival [21,22]. Furthermore, the application of machine learning techniques and deep clustering has allowed the derivation of refined AKI subphenotypes, identifying seven subgroups defined by comorbidities, laboratory parameters, and mortality [23]; this stratification has been further confirmed in recent systematic reviews [24].

Those studies have largely relied on advanced methodologies, such as molecular biomarkers, deep clustering, or creatinine trajectory modeling, applied in specific contexts like sepsis, cardiac surgery, or critically ill populations. However, they often require specialized assays, complex computational tools, and are not always feasible for routine clinical implementation. In contrast, our study focuses on the identification of biochemical phenotypes using readily available laboratory parameter**s** including hemoglobin, calcium, phosphorus, potassium, and pH in a broad cohort of hospitalized patients with AKI. This pragmatic approach allowed the derivation of clinically interpretable clusters that capture the systemic metabolic spectrum of AKI and can be applied in real time at the bedside. Importantly, the biochemical phenotypes identified in this cohort were strongly associated with MAKE, providing a simple yet powerful tool for risk stratification beyond traditional KDIGO staging. This approach not only complements existing molecular and trajectory-based classifications but also bridges the gap between research-driven phenotyping and real-world clinical applicability, where early identification of high-risk subgroups may guide therapeutic decisions and resource allocation.

The pathophysiology underlying the poorer clinical trajectory of Phenotype 3 may be explained by the characteristics that distinguish it from the other AKI groups. This phenotype was characterized by higher levels of creatinine, phosphate, and potassium, together with more severe metabolic acidosis. Each of these abnormalities independently has robust evidence linking it to MAKE: for example, elevated phosphate levels have been associated with worse prognosis and increased need for KRT in critically ill patients with AKI [25], while persistent metabolic acidosis has been related to adverse clinical courses, including progression to CKD [26]. It is therefore intuitive to consider that when these derangements coexist in the same patient, they may act synergistically to create a metabolically unstable environment that precipitates severe complications. Phenotype 2, in contrast, may represent an intermediate stage in the transition toward Phenotype 3. Anemia in this group could result from gastrointestinal blood loss, bone marrow infiltration by malignancy, persistent systemic inflammation, or deficiencies in iron and erythropoietin [27,28]. Hypocalcemia, on the other hand, may be explained by hypoalbuminemia, vitamin D deficiency, inhibition of parathyroid hormone secretion, or calcium sequestration within bone in inflammatory states [29]. The observed associations with gastrointestinal bleeding and cancer suggest that these conditions may serve as triggers for metabolic decompensation, predisposing patients to progressive biochemical deterioration.

The hypothesis that Phenotype 2 represents an intermediate state to Phenotype 3 is consistent with the existing literature on risk stratification in AKI. Phenotypic classification studies, have identified subgroups with partial biochemical abnormalities that, in the presence of additional physiological insults, progress toward high-mortality profiles [30]. From a pathophysiological standpoint, it is plausible that Phenotype 2 reflects a state of diminished physiological reserve, in which the occurrence of a second precipitating event, such as sepsis or acute cardiac decompensation may drive progression toward a pattern of severe metabolic disturbance. Such progression could potentially be captured through dynamic markers, including rising phosphate and potassium levels and worsening acidosis, which correlate with increases in composite severity scores, as evaluated in the present study.

Early recognition of patients with Phenotype 2 may have direct clinical implications. Unlike Phenotype 1, these patients already exhibit two cardinal abnormalities anemia and hypocalcemia both of which are established predictors of poor outcomes in AKI and chronic kidney disease [2,10]. Addressing these abnormalities through hemoglobin optimization, vitamin D supplementation, correction of hypocalcemia, and management of the underlying cause may help delay or even prevent progression to a more severe clinical state. However, this remains an area of growing interest with limited clinical and therapeutic evidence. The clinical applicability of this phenotypic classification lies in its reliance on routine biochemical variables, which are accessible even in resource-limited hospitals. This approach enables real-time risk stratification and can guide decisions regarding monitoring intensity, early initiation of supportive measures, or timely referral to nephrology. Integration of such classification into electronic alert systems, in combination with predictive models, could further enhance the management and outcomes of patients with AKI.

A key strength of this study is the large cohort size, which enhances the robustness of the analyses, together with the use of an unsupervised clustering approach that minimizes bias from predefined classifications. The internal validation through multivariable modeling further supports the reliability of the findings. These strengths underscore the potential clinical utility of this classification, as it is based on routinely available biochemical variables that could be readily implemented in diverse hospital settings.

However, several limitations warrant consideration when translating these results into practice. The retrospective design restricts causal inference, and the absence of longitudinal follow-up prevents confirmation of whether Phenotype 2 truly evolves into the higher-risk Phenotype 3. Adjustment for additional factors such as medication exposure and markers of multi-organ failure was limited by data availability and therefore not included in the primary models.

In addition, the lack of emerging biomarkers may have limited the refinement of phenotypic definitions, potentially underestimating the heterogeneity of AKI. The lack of formal cluster stability and external validation analyses represents a limitation and may affect the reproducibility of the identified phenotypes. In addition, follow-up was limited to the in-hospital period, which precludes evaluation of long-term outcomes and may introduce time-related bias.

Finally, as this was a single-center study, external validation is necessary before generalization. Future multicenter and prospective studies, ideally incorporating biomarker data and dynamic patient monitoring, will be essential to determine whether this phenotypic classification can be integrated into clinical decision-making and electronic alert systems to improve outcomes in AKI.

## Conclusion

Routine biochemical phenotyping in AKI enables the identification of clinically meaningful subgroups. Phenotype 3 reflects critical metabolic instability with high risk of adverse outcomes, while Phenotype 2 may represent an intermediate stage where timely intervention could alter disease trajectory. Prospective multicenter validation is required to confirm these findings and guide their integration into clinical practice.

## Supplementary Material

Supplementary Appendix .docx

## Data Availability

The files and data are in the physical and electronic archives of the Hospital General de Mazatlán and can be requested with prior authorization. All data generated or analyzed during this study are included in this article. Further inquiries can be directed to the corresponding author.
