# Supplementary material for: Biochemical phenotypes of acute kidney injury and their association with major adverse kidney events
Source: Ren Fail. 2026 Jan 27;48(1):2620162. doi: 10.1080/0886022X.2026.2620162 (PMC12854221; doi:10.1080/0886022X.2026.2620162)
Supplement: Supplementary Appendix .docx [file IRNF_A_2620162_SM1603.docx]

# Supplementary Appendix - Biochemical Phenotypes of Acute Kidney Injury and Their Association with Major Adverse Kidney Events

Supplementary logistic regression analyses supporting the robustness of the composite outcome (Major Adverse Kidney Events, MAKE). Results are presented as odds ratios (OR) with 95% confidence intervals.

## Supplementary Table A1. Separate Multivariable Logistic Regression Models for MAKE Components

### A1a. In-hospital mortality

| Variable | OR | 2.5% CI | 97.5% CI |
| --- | --- | --- | --- |
| Intercept | 0.0238958 | 0.008683195 | 0.06291352 |
| Phenotype 3 (Cluster 3) | 2.5482877 | 1.541455606 | 4.23083479 |
| Phenotype 2 (Cluster 2) | 1.4461006 | 0.952611717 | 2.19947020 |
| Age (years) | 1.0300792 | 1.017439473 | 1.04324369 |
| Female sex | 0.8789860 | 0.607575561 | 1.26798842 |
| AKI stage 2 (vs stage 1) | 1.3409172 | 0.828226499 | 2.16018793 |
| AKI stage 3 (vs stage 1) | 1.1082098 | 0.693079799 | 1.76775310 |
| Septic shock | 4.9690464 | 3.164090765 | 7.87729799 |
| Pancreatitis | 0.3829668 | 0.015052973 | 6.29437379 |
| Urinary tract infection | 0.8291669 | 0.532394605 | 1.27250904 |
| Acute coronary syndrome | 1.3173823 | 0.571341550 | 2.84528160 |
| Acute heart failure | 1.1365696 | 0.651501637 | 1.93399696 |
| Gastrointestinal bleeding | 1.5391539 | 0.847607792 | 2.75361798 |
| COPD exacerbation | 0.5509792 | 0.248890122 | 1.14061384 |
| Community-acquired pneumonia | 1.2027538 | 0.869576578 | 1.67620190 |
| Hypertensive emergency | 0.6609436 | 0.255607731 | 1.49955830 |
| Malignancy | 4.8729392 | 2.128919165 | 11.50155850 |
| Decompensated cirrhosis | 2.8436995 | 1.387601789 | 5.75334959 |
| Cerebrovascular event | 1.8614771 | 0.938182363 | 3.63139220 |
| Seizure disorder | 1.2872114 | 0.420306034 | 3.48238104 |
| Tuberculosis | 1.4818028 | 0.560837350 | 3.67512394 |
| Diabetic ketoacidosis | 0.1617825 | 0.025007300 | 0.58668693 |
| Hemodialysis | 0.3050253 | 0.067264205 | 0.98433666 |

### A1b. Initiation of kidney replacement therapy (parsimonious model)

| **Variable** | **OR** | **2.5% CI** | **97.5% CI** |
| --- | --- | --- | --- |
| Intercept | 8.302247e-10 | 2.641599e-100 | 2.259462e+22 |
| Phenotype 3 (Cluster 3) | 5.827248e+00 | 1.652396e+00 | 3.050407e+01 |
| Phenotype 2 (Cluster 2) | 8.774004e-01 | 1.644918e-01 | 5.573009e+00 |
| Age (years) | 9.806428e-01 | 9.508392e-01 | 1.010023e+00 |
| Female sex | 1.096841e+00 | 4.363777e-01 | 2.688759e+00 |
| AKI stage 2 | 6.942125e-01 | 1.391109e-24 | 3.464366e+23 |
| AKI stage 3 | 1.247087e+08 | 1.350660e-23 | 6.159738e+130 |
| Septic shock | 7.452226e-01 | 1.439239e-01 | 2.748663e+00 |
| Pancreatitis | 9.638710e+00 | 3.056182e-01 | 3.152059e+02 |
| Urinary tract infection | 8.140721e-01 | 2.402712e-01 | 2.346680e+00 |
| Acute coronary syndrome | 8.534819e+00 | 3.014506e-01 | 1.633024e+02 |
| Acute heart failure | 1.213041e+00 | 2.991344e-01 | 4.021079e+00 |
| Gastrointestinal bleeding | 2.769941e+00 | 5.560972e-01 | 1.089247e+01 |
| COPD exacerbation | 1.007705e-07 | 2.165269e-293 | 1.258925e+65 |
| Community-acquired pneumonia | 1.675553e+00 | 2.224959e-01 | 8.206554e+00 |
| Hypertensive emergency | 1.771049e+00 | 4.323299e-01 | 6.143479e+00 |
| Malignancy | 1.755400e+00 | 8.246897e-02 | 1.464837e+01 |
| Decompensated cirrhosis | 1.502556e-08 | 0.000000e+00 | 6.507272e+73 |
| Cerebrovascular event | 3.377195e-08 | 2.176036e-228 | 1.030931e+69 |
| Seizure disorder | 8.928445e-09 | 0.000000e+00 | 3.942924e+94 |
| Tuberculosis | 2.785469e-08 | 0.000000e+00 | 8.899675e+96 |
| Diabetic ketoacidosis | 1.716042e+00 | 1.973870e-01 | 9.346052e+00 |

### A1c. Major Adverse Kidney Events (MAKE)

| **Variable** | **OR** | **2.5% CI** | **97.5% CI** |
| --- | --- | --- | --- |
| Intercept | 0.03698759 | 0.01441675 | 0.09155394 |
| Phenotype 3 (Cluster 3) | 3.37941567 | 2.11153941 | 5.45152365 |
| Phenotype 2 (Cluster 2) | 1.35471585 | 0.90241484 | 2.03587158 |
| Age (years) | 1.02455920 | 1.01271734 | 1.03682061 |
| Female sex | 0.90853787 | 0.63873192 | 1.28971909 |
| AKI stage 2 | 1.21346561 | 0.75361971 | 1.94169487 |
| AKI stage 3 | 1.37308772 | 0.88134203 | 2.13977262 |
| Septic shock | 4.13175047 | 2.65493041 | 6.48092456 |
| Pancreatitis | 2.37291098 | 0.15352789 | 33.81387330 |
| Urinary tract infection | 0.79748919 | 0.52032056 | 1.20621867 |
| Acute coronary syndrome | 1.43727824 | 0.64556861 | 3.03588669 |
| Acute heart failure | 0.99794437 | 0.58143037 | 1.67460617 |
| Gastrointestinal bleeding | 1.53821505 | 0.86424448 | 2.70806805 |
| COPD exacerbation | 0.54787964 | 0.24856251 | 1.12954809 |
| Community-acquired pneumonia | 1.17666019 | 0.85046786 | 1.62424565 |
| Hypertensive emergency | 0.89406146 | 0.40778115 | 1.83187344 |
| Malignancy | 4.88520434 | 2.13985164 | 11.69602181 |
| Decompensated cirrhosis | 2.56415138 | 1.25836266 | 5.15584611 |
| Cerebrovascular event | 1.74122367 | 0.88285207 | 3.37828796 |
| Seizure disorder | 1.08977735 | 0.36101564 | 2.90076245 |
| Tuberculosis | 1.33063761 | 0.51300280 | 3.24427589 |
| Diabetic ketoacidosis | 0.27950709 | 0.07767984 | 0.78083510 |

Sensitivity analyses using separate multivariable logistic regression models for each component of MAKE demonstrated consistent and directionally concordant associations. Phenotype 3 was independently associated with both in-hospital mortality (OR ≈2.5) and initiation of kidney replacement therapy (OR ≈5.8), supporting the robustness of the composite outcome. As expected given the low number of KRT events, confidence intervals were wider in KRT models; however, the magnitude and direction of the association for phenotype 3 were consistent across all outcomes.

### ****Supplementary Table A2a. Multivariable Logistic Regression Model for MAKE in Patients with De Novo AKI (Without Pre-existing CKD)****

This table presents a multivariable logistic regression model evaluating factors associated with major adverse kidney events (MAKE) among patients with acute kidney injury (AKI) without pre-existing chronic kidney disease (CKD). Results are reported as odds ratios (OR) with corresponding 95% confidence intervals.

| **Variable** | **OR** | **2.5% CI** | **97.5% CI** |
| --- | --- | --- | --- |
| Intercept | 0.02141774 | 0.006956651 | 0.06233919 |
| Phenotype 3 (Cluster 3) | 3.35768477 | 1.932264091 | 5.88314562 |
| Phenotype 2 (Cluster 2) | 1.63652475 | 1.030334617 | 2.60691197 |
| Age (years) | 1.03108038 | 1.017150991 | 1.04567003 |
| Female sex | 0.90753816 | 0.596611087 | 1.37484914 |
| AKI stage 2 | 1.25291097 | 0.732627216 | 2.12722502 |
| AKI stage 3 | 1.92505122 | 1.135844443 | 3.27186721 |
| Septic shock | 4.89875247 | 2.893757431 | 8.42470241 |
| Pancreatitis | 4.59390779 | 0.067454587 | 261.63848850 |
| Urinary tract infection | 0.75921728 | 0.454406631 | 1.24510342 |
| Acute coronary syndrome | 1.34941087 | 0.518505651 | 3.25458011 |
| Acute heart failure | 1.09321474 | 0.553732918 | 2.08554611 |
| Gastrointestinal bleeding | 1.35775387 | 0.697120492 | 2.60590459 |
| COPD exacerbation | 0.63893728 | 0.275161279 | 1.39421842 |
| Community-acquired pneumonia | 1.16604792 | 0.813021616 | 1.63236460 |
| Hypertensive emergency | 1.43178659 | 0.578686418 | 3.31583132 |
| Malignancy | 7.32103425 | 2.738305473 | 21.92510529 |
| Decompensated cirrhosis | 2.97526630 | 1.333740383 | 6.54306330 |
| Cerebrovascular event | 1.69419413 | 0.810253018 | 3.48033645 |
| Seizure disorder | 0.96267789 | 0.281153386 | 2.79308073 |
| Tuberculosis | 1.37291767 | 0.500674526 | 3.57722212 |
| Diabetic ketoacidosis | 0.16023627 | 0.023520012 | 0.62678396 |

### ****Supplementary Table A2b. Multivariable Logistic Regression Model for MAKE in Patients with AKI on Pre-existing Chronic Kidney Disease****

This table presents a multivariable logistic regression model assessing factors associated with major adverse kidney events (MAKE) among patients with acute kidney injury (AKI) and pre-existing chronic kidney disease (CKD). Results are reported as odds ratios (OR) with corresponding 95% confidence intervals.

| **Variable** | **OR** | **2.5% CI** | **97.5% CI** |
| --- | --- | --- | --- |
| Intercept | 4.920975e-02 | 0.004969828 | 4.089281e-01 |
| Phenotype 3 (Cluster 3) | 3.469765e+00 | 1.282052074 | 1.005770e+01 |
| Phenotype 2 (Cluster 2) | 1.095099e+00 | 0.422529349 | 2.895228e+00 |
| Age (years) | 1.014145e+00 | 0.988122037 | 1.041711e+00 |
| Female sex | 1.054996e+00 | 0.491483763 | 2.291511e+00 |
| AKI stage 2 | 1.743537e+00 | 0.484832879 | 6.419804e+00 |
| AKI stage 3 | 1.429873e+00 | 0.468300361 | 4.726380e+00 |
| Septic shock | 2.606367e+00 | 1.049938674 | 6.496837e+00 |
| Pancreatitis | 1.734204e+00 | 0.043987033 | 7.043505e+01 |
| Urinary tract infection | 9.667264e-01 | 0.401925571 | 2.225325e+00 |
| Acute coronary syndrome | 2.184027e+00 | 0.382292207 | 1.102554e+01 |
| Acute heart failure | 1.538821e+00 | 0.545861644 | 4.242736e+00 |
| Gastrointestinal bleeding | 2.074194e+00 | 0.590194581 | 6.961755e+00 |
| Community-acquired pneumonia | 1.489781e+00 | 0.473313684 | 4.414295e+00 |
| Hypertensive emergency | 1.603658e-01 | 0.008075794 | 9.925378e-01 |
| Malignancy | 1.507716e+00 | 0.174200633 | 1.075328e+01 |
| Decompensated cirrhosis | 1.982256e+00 | 0.353539489 | 1.027929e+01 |
| Cerebrovascular event | 1.615329e+00 | 0.170242427 | 1.229982e+01 |
| Diabetic ketoacidosis | 7.866376e-01 | 0.098060765 | 4.330711e+00 |

### ****Supplementary Table A2c. Formal Interaction Analysis Between AKI Phenotype and Pre-existing Chronic Kidney Disease for MAKE****

This table presents the results of a formal interaction analysis evaluating whether the association between AKI phenotypes and major adverse kidney events (MAKE) differs according to the presence of pre-existing chronic kidney disease (CKD). A multivariable logistic regression model including an interaction term between phenotype and CKD status was fitted. Results are shown as regression coefficients (β), standard errors, z values, and corresponding p values.

| **Variable** | **Estimate (β)** | **Std. Error** | **z value** | **p value** |
| --- | --- | --- | --- | --- |
| Intercept | -3.444606 | 0.481147 | -7.159 | 8.12e-13 |
| Phenotype 3 (Cluster 3) | 1.205472 | 0.275219 | 4.380 | 1.19e-05 |
| Phenotype 2 (Cluster 2) | 0.478756 | 0.230167 | 2.080 | 0.037522 |
| Chronic kidney disease (CKD) | -0.199446 | 0.385856 | -0.517 | 0.605231 |
| Age (years) | 0.025620 | 0.006054 | 4.232 | 2.32e-05 |
| Female sex | -0.052747 | 0.181109 | -0.291 | 0.770863 |
| AKI stage 2 | 0.248741 | 0.243361 | 1.022 | 0.306730 |
| AKI stage 3 | 0.505600 | 0.239506 | 2.111 | 0.034771 |
| Septic shock | 1.447963 | 0.229445 | 6.311 | 2.78e-10 |
| Pancreatitis | 0.855952 | 1.445960 | 0.592 | 0.553876 |
| Urinary tract infection | -0.245828 | 0.216403 | -1.136 | 0.255967 |
| Acute coronary syndrome | 0.415660 | 0.395365 | 1.051 | 0.293106 |
| Acute heart failure | 0.078597 | 0.272422 | 0.289 | 0.772955 |
| Gastrointestinal bleeding | 0.425662 | 0.292962 | 1.453 | 0.146235 |
| COPD exacerbation | -0.609254 | 0.386780 | -1.575 | 0.115212 |
| Community-acquired pneumonia | 0.163549 | 0.149788 | 1.092 | 0.274892 |
| Hypertensive emergency | -0.060241 | 0.382780 | -0.157 | 0.874946 |
| Malignancy | 1.613693 | 0.434779 | 3.712 | 0.000206 |
| Decompensated cirrhosis | 0.992672 | 0.358392 | 2.770 | 0.005609 |
| Cerebrovascular event | 0.523411 | 0.343504 | 1.524 | 0.127573 |
| Seizure disorder | 0.070584 | 0.524333 | 0.135 | 0.892916 |
| Tuberculosis | 0.203633 | 0.471584 | 0.432 | 0.665882 |
| Diabetic ketoacidosis | -1.321455 | 0.581308 | -2.273 | 0.023011 |
| Phenotype 3 × CKD | -0.048092 | 0.524887 | -0.092 | 0.926998 |
| Phenotype 2 × CKD | -0.698441 | 0.510524 | -1.368 | 0.171285 |

We performed additional sensitivity analyses stratified by baseline chronic kidney disease (CKD) status. In patients with de novo AKI (without CKD), phenotype 3 remained strongly associated with MAKE (OR 3.36, 95% CI 1.93–5.88). Importantly, a similar magnitude of association was observed among patients with AKI on CKD (OR 3.47, 95% CI 1.28–10.06), despite wider confidence intervals due to smaller subgroup size. Formal interaction testing showed no significant phenotype–CKD interaction (p for interaction = 0.17), indicating that the association between phenotype 3 and MAKE was consistent regardless of baseline CKD status.

### ****Supplementary Table A3a. Multivariable Logistic Regression Model for MAKE in Non-Septic Acute Kidney Injury****

This table shows a multivariable logistic regression model evaluating factors associated with major adverse kidney events (MAKE) among patients with non-septic acute kidney injury (AKI). Results are expressed as odds ratios (OR) with corresponding 95% confidence intervals*.*

| **Variable** | **OR** | **2.5% CI** | **97.5% CI** |
| --- | --- | --- | --- |
| Intercept | 0.05317864 | 0.014837832 | 0.1793715 |
| Phenotype 3 (Cluster 3) | 2.96816678 | 1.593239069 | 5.5914368 |
| Phenotype 2 (Cluster 2) | 1.19549322 | 0.678202210 | 2.1060370 |
| Age (years) | 1.01941693 | 1.003211902 | 1.0363252 |
| Female sex | 0.96076723 | 0.594033092 | 1.5461298 |
| Chronic kidney disease (CKD) | 0.65004412 | 0.363872991 | 1.1360602 |
| AKI stage 2 | 1.33054232 | 0.679654188 | 2.5643078 |
| AKI stage 3 | 1.96680419 | 1.026344997 | 3.7906192 |
| Pancreatitis | 16.77836274 | 0.556524269 | 551.6745188 |
| Acute coronary syndrome | 1.56752103 | 0.527882696 | 4.1551137 |
| Acute heart failure | 0.79983599 | 0.388614268 | 1.5669996 |
| Gastrointestinal bleeding | 1.96782664 | 0.942605368 | 4.0606740 |
| Hypertensive emergency | 1.03390214 | 0.444881601 | 2.2463254 |
| Malignancy | 8.69422946 | 2.697899990 | 33.7513397 |
| Decompensated cirrhosis | 1.91175399 | 0.771355746 | 4.5364938 |
| Cerebrovascular event | 1.37374932 | 0.544001341 | 3.2675890 |
| Seizure disorder | 1.24727011 | 0.291418911 | 4.4164768 |
| Diabetic ketoacidosis | 0.09624191 | 0.003772754 | 0.6042461 |

### ****Supplementary Table A3b. Multivariable Logistic Regression Model for MAKE in Septic Acute Kidney Injury****

This table presents a multivariable logistic regression model evaluating factors associated with major adverse kidney events (MAKE) among patients with septic acute kidney injury (AKI). Results are reported as odds ratios (OR) with corresponding 95% confidence intervals.

| **Variable** | **OR** | **2.5% CI** | **97.5% CI** |
| --- | --- | --- | --- |
| Intercept | 0.02832507 | 0.006903532 | 0.1066892 |
| Phenotype 3 (Cluster 3) | 4.21411436 | 2.073168447 | 8.7445628 |
| Phenotype 2 (Cluster 2) | 1.96448536 | 1.099022426 | 3.5512724 |
| Age (years) | 1.03363705 | 1.016691453 | 1.0517305 |
| Female sex | 0.86769186 | 0.517649081 | 1.4500095 |
| Chronic kidney disease (CKD) | 0.63460215 | 0.329112990 | 1.1932592 |
| AKI stage 2 | 1.60340683 | 0.822203694 | 3.1302760 |
| AKI stage 3 | 1.33918750 | 0.686396433 | 2.6129253 |
| Pancreatitis | 0.94377898 | 0.036078975 | 24.6669031 |
| Acute coronary syndrome | 0.96319377 | 0.300587343 | 2.8428563 |
| Acute heart failure | 1.43173312 | 0.609034468 | 3.2901717 |
| Gastrointestinal bleeding | 1.14602348 | 0.465507278 | 2.7357033 |
| Hypertensive emergency | 0.27885992 | 0.014334035 | 1.7023459 |
| Malignancy | 2.15714938 | 0.661322095 | 7.2342590 |
| Decompensated cirrhosis | 3.63857128 | 1.088695011 | 13.3695762 |
| Cerebrovascular event | 1.99574931 | 0.731631846 | 5.5538642 |
| Seizure disorder | 0.62507139 | 0.089759119 | 2.7208575 |
| Diabetic ketoacidosis | 0.54025016 | 0.110725570 | 2.0208097 |

### ****Supplementary Table A3c. Formal Interaction Analysis Between AKI Phenotype and Septic Etiology for MAKE****

This table presents the results of a formal interaction analysis assessing whether the association between AKI phenotypes and major adverse kidney events (MAKE) differs according to AKI etiology (septic vs non-septic). A multivariable logistic regression model including an interaction term between phenotype and septic AKI was fitted. Results are reported as odds ratios (OR) with corresponding 95% confidence intervals.

| **Variable** | **OR** | **2.5% CI** | **97.5% CI** |
| --- | --- | --- | --- |
| Intercept | 0.04087718 | 0.01616030 | 0.09976175 |
| Phenotype 3 (Cluster 3) | 3.32820472 | 1.85129855 | 6.03997460 |
| Phenotype 2 (Cluster 2) | 1.31287216 | 0.76515570 | 2.25626149 |
| Septic AKI (vs non-septic AKI) | 1.28795318 | 0.75190593 | 2.20862218 |
| Age (years) | 1.02420247 | 1.01281978 | 1.03600684 |
| Female sex | 0.93718957 | 0.66457989 | 1.31947316 |
| Chronic kidney disease (CKD) | 0.64366436 | 0.41910895 | 0.97663075 |
| AKI stage 2 | 1.43232745 | 0.90485675 | 2.25799744 |
| AKI stage 3 | 1.64987989 | 1.04871759 | 2.59970605 |
| Pancreatitis | 3.69358527 | 0.32779633 | 38.10548203 |
| Acute coronary syndrome | 1.30468665 | 0.60214072 | 2.68970822 |
| Acute heart failure | 0.94247207 | 0.55467497 | 1.56565948 |
| Gastrointestinal bleeding | 1.58430294 | 0.90645648 | 2.74534976 |
| Hypertensive emergency | 0.83651954 | 0.38518287 | 1.69417061 |
| Malignancy | 4.32056564 | 1.91837035 | 10.23085589 |
| Decompensated cirrhosis | 2.30447003 | 1.14913351 | 4.56935963 |
| Cerebrovascular event | 1.69328466 | 0.88306411 | 3.20340101 |
| Seizure disorder | 0.97115838 | 0.33067442 | 2.50732767 |
| Diabetic ketoacidosis | 0.27486807 | 0.07705447 | 0.75726963 |
| Phenotype 3 × Septic AKI | 1.06528342 | 0.45087435 | 2.52536914 |
| Phenotype 2 × Septic AKI | 1.22472024 | 0.57133948 | 2.63024911 |

Sensitivity analyses stratified by AKI etiology were performed using a clinically contextual classification into septic and non-septic AKI. In patients with non-septic AKI, phenotype 3 remained independently associated with MAKE (OR 2.97, 95% CI 1.59–5.59), whereas phenotype 2 showed no statistically significant association. Similarly, among patients with septic AKI, phenotype 3 demonstrated an even stronger association with MAKE (OR 4.21, 95% CI 2.07–8.74), while phenotype 2 showed a more modest effect.

Formal interaction testing revealed no significant interaction between AKI etiology and phenotype 3 (OR for interaction 1.22, 95% CI 0.57–2.63), indicating that the association between phenotype 3 and MAKE was consistent across septic and non-septic AKI contexts.
